# Inspecting mother-to-infant microbiota transmission: disturbance of strain inheritance by cesarian section

**DOI:** 10.3389/fmicb.2024.1292377

**Published:** 2024-02-29

**Authors:** Ru Yang, Yinan Wang, Zhiye Ying, Zeyao Shi, Yan Song, Jing Yan, Shulin Hou, Zicheng Zhao, Yanling Hu, Qiong Chen, Wentao Peng, Xiaowen Li

**Affiliations:** ^1^Department of Neonatology Nursing, West China Second University Hospital, Sichuan University, Chengdu, China; ^2^Key Laboratory of Birth Defects and Related Diseases of Women and Children, Sichuan University, Ministry of Education, Chengdu, China; ^3^Department of Obstetrics and Gynecology, Peking University Shenzhen Hospital, Shenzhen, China; ^4^West China Biomedical Big Data Center, West China Hospital, Sichuan University, Sichuan, China; ^5^Medical Big Data Center, Sichuan University, Chengdu, Sichuan, China; ^6^Shenzhen Byoryn Technology, Shenzhen, Guangdong, China

**Keywords:** mothers, infants, metagenomics, gut microbiome, strain transmission, cesarean section

## Abstract

**Introduction:**

The initial acquisition and subsequent development of the microbiota in early life is crucial to future health. Cesarean-section (CS) birth is considered to affect early microbial transmission from mother to infant.

**Methods:**

In this study, we collected fecal samples from 34 CS infants and their mothers from West China Second Hospital, Sichuan University to assess the microbiota developmental trajectory of mothers and infants. We explored mother-infant gut microbiome transmission via comparison with corresponding Finnish data.

**Results:**

Metagenomic analysis of gut microbiota profiles indicated that the communities of mothers and infants were distinct. The composition of the infant gut microbiome was highly variable but also followed predictable patterns in the early stages of life. Maternal communities were stable and mainly dominated by species from *Bacteroidacea* spp. We used PStrain to analyze and visualize strain transmission in each mother-infant pair. Excluding missing data, we included 32 mother-infant pairs for analysis of strain transmission. Most CS deliveries (65.6%, 21/32) did not demonstrate transmission of strains from mother to infant. To further explore the mother-infant strain transmission, we analyzed metagenomics data from Finnish mother-infant pairs. A total of 32 mother-infant pairs were included in the analysis, including 28 vaginal delivery (VD) infants and four CS infants. Strain transmission was observed in 30 infants, including 28 VD infants and two CS infants. All VD infants received transmitted stains from their mothers. Finally, a total of 193 strain transmission events were observed, comprising 131 strains and 45 species.

**Discussion:**

Taken together, our data suggested that delivery mode was an important factor influencing the mother-infant strain transmission.

## Introduction

The human gut microbiome is known to be involved in the development of the immune system, host metabolism, and colonization resistance to enteric pathogens (Buffie and Pamer, 2013; Wopereis et al., 2014; Kerr et al., 2015). The initial acquisition and subsequent development of the microbiota are crucial processes for the host, ultimately affecting the host’s health in the long term. The delivery mode strongly influences the early development of infant gut microbiome. Cesarean section (CS) infants have significantly different early gut microbial communities compared to vaginal delivery (VD) infants, including lower microbiota diversity and less colonization with the *Bacteroides* species (Jakobsson et al., 2014; Mitchell et al., 2020; Matharu et al., 2022). CS is associated with the perturbation and delayed maturation of the gut microbiota (Stokholm et al., 2016; Long et al., 2021). The early alteration of gut microbiota in CS infants may induce intestinal inflammation and alter the epithelium structure and the mucus-producing cells, thereby disrupting the gut homeostasis (Barone et al., 2023). The differences in gut microbiome due to delivery mode gradually decrease with age. However, these differences persist up to 4 years of age (Fouhy et al., 2019). A perturbed gut microbiota in this critical window may cause lifelong influences. Children born by CS are associated with short- and long-term effects, including an increase in the risk of asthma (Stokholm et al., 2020; Gürdeniz et al., 2022), and chronic immune or inflammatory diseases (Keag et al., 2018; Sandall et al., 2018; Andersen et al., 2020).

After birth, infants are continuously exposed to multiple microbiome communities, including those of mothers (Yassour et al., 2018; Bogaert et al., 2023), family members (Korpela et al., 2018; Hildebrand et al., 2021), and the hospital environment (Raveh-Sadka et al., 2016; Brooks et al., 2017). Among the multiple microbiome sources, the maternal microbiota is an important microbiome reservoir for neonate colonization and a source of continual exposure to infants in early life (Ferretti et al., 2018). A total of 58.5% of the infant microbiota composition can be attributed to any of the maternal source communities (Bogaert et al., 2023). Studies have investigated the microbiome transmission from mother to infant across multiple body sites, focusing on possible maternal sources such as maternal gut (Shao et al., 2019; Mitchell et al., 2020), vagina (Mortensen et al., 2021; Song et al., 2021), and breastmilk (Pannaraj et al., 2017; Fehr et al., 2020) microbiota. Previously, it was widely believed that the microbes of VD infants were transmitted from the maternal vaginal microbiome. Maternal vaginal strains rarely colonize the infant’s gut. Evidence revealed that the maternal gut microbiome is the main source of the transmitted strains, with partial strains transferred from the maternal oral cavity and vagina (Mitchell et al., 2020; Xiao and Zhao, 2023). Strain-level metagenomic profiling showed that the maternal gut microbiome accounted for 22.1% of the overall microbial abundance in the infant’s gut, followed by vaginal (16.3%), oral (7.2%), and skin (5%) microbiome (Ferretti et al., 2018). Meanwhile, strains transmitted from the gut are highly stable and persist for at least 1 year, whereas the strains transmitted from maternal skin and vagina only undergo transient colonization (Ferretti et al., 2018; Korpela et al., 2018).

Cesarean-section disrupts the patterns of microbiota transmission from mother to infant and delays microbiota maturation in the first year. *Bacteroides* spp., *Parabacteroides* spp., *E. coli*, and *Bifidobacterium* spp. are most frequently transmitted from mothers to babies through vaginal birth (Wampach et al., 2018; Shao et al., 2019). Instead, CS infants are often associated with a lack of transmission of maternal enteric *Bacteroides* strains (Korpela et al., 2018; Shao et al., 2019; Wang et al., 2021b). CS infants generally experience colonization by opportunistic pathogens such as *Enterococcus* and *Klebsiella* species originating from hospital environments (Shao et al., 2019). Transferred strains that are lacking in CS infants are involved in important microbial pathways such as lipopolysaccharide biosynthesis and may be relevant in immune stimulation potential during the critical window profoundly influencing the health of infants in later life (Wampach et al., 2018).

A range of studies have investigated mother-infant microbiome transmission with different focuses. However, limited studies have extensively analyzed strain transmission events within individual maternal-infant pairs. In this study, we performed metagenomic analysis to determine the microbiota development trajectory of CS infants and their mothers in early life and used PStrain to explore mother-infant strain transmission. We quantified and visualized the strain transmission events in each maternal-infant pair during the first 3 months.

## Materials and methods

### Subject recruitment and sample collection

This study obtained approval from the Ethics Committee of West China Second Hospital, Sichuan University (Approval No. 2021112). Written informed consent was obtained from all participants. A total of 34 mother-infant pairs were recruited from December 2020 to December 2021 at West China Second Hospital, Sichuan University. Mothers with metabolic disorders such as obesity, diabetes, hypertension, and hyperuricemia, as well as mothers with immune-related diseases such as rheumatoid arthritis, lupus erythematosus, Hashimoto’s thyroiditis, and ulcerative colitis, were excluded from the study. Additionally, mothers who declined to participate or withdrew from the study were also excluded. All infants were delivered by C-section. Prior to sample collection, we provided detailed explanations to the mothers regarding the precautions for sample collection. Maternal fecal samples were collected both in the hospital and at home within the last week of pregnancy and 1 month postpartum. Infant samples were collected 1 week and 3 months after birth. Fecal samples collected in the hospital were initially frozen at −20°C in the laboratory and then transferred to −80°C within 24 h. Maternal and infant fecal samples collected at home were shipped to the laboratory at West China Second Hospital of Sichuan University within 2–3 days and stored at −80°C. According to the instructions of the fecal collection tube (Shbio scRNA-seq Kit, Shanghai, China), the samples can be stored at room temperature for 14 days.^1^

### Fecal genomic DNA extraction and sequencing

The DNA extraction from the samples was performed using the QIAamp PowerFecal Pro DNA Kit (QIAGEN, 51804, USA) following the manufacturer’s instructions. The extracted DNA concentration was measured using a NanoDrop spectrophotometer (Thermo Fisher Scientific, USA) and a Qubit fluorometer (Invitrogen, USA). All extracted DNA samples were stored at −80°C until further processing for shotgun metagenomic sequencing. Negative controls were included using the same procedures as the actual samples. DNA was randomly sheared using Covaris, and magnetic bead selection was employed to isolate DNA fragments with an average size of 200–400 bp. The selected fragments underwent end repair, 3’ adenylation, adapter ligation, and PCR amplification, followed by purification using magnetic beads. The double-stranded PCR products were denatured, and circularization was performed using splint oligonucleotides. The single-stranded circular DNA (ssCir DNA) was formatted into the final library and underwent quality control. Paired-end sequencing was performed on the DNBSEQ-T7 platform (BGI, Shenzhen, China), with an insert size of 350 bp and paired-end (PE) reads of 150 bp. The target sequence for each sample was approximately 10 Gb.

### Quality control

Quality control of the sequencing data was performed using KneadData^2^ with default parameters. Adapters were removed, and reads with low-quality bases were trimmed (windows: 4-mer, Phred quality < 20) and truncated (<50% of pre-trimmed length) using Trimmomatic (version = 0.39). To eliminate potential human contamination, the filtered paired-end reads were mapped to the human genome (hg19, GCA_000001405.1) using bowtie2 (version = 2.3.1) with default parameters (–very-sensitive).

### Taxonomy and functional annotations

We employed the wmgx bioBakery workflow to perform taxonomic annotation and functional analysis of metagenomic sequencing data^3^ (Beghini et al., 2021). Taxonomic annotation was performed using the default parameters of MetaPhlAn3 (version = 3.0.14). Functional analyses were performed using the default parameters of HUMAnN3 (version = 3.0.1). The CHOCOPhlAn (release 2019.01) database was used for taxonomic and functional annotation, and the UniRef90 database (version 2021.03) was used to determine gene family abundance.

### Bioinformatic analysis

We utilized the ImageGP to visually display the abundance and evenness of the microbiome composition using the Shannon index. The differences between two groups were assessed using the Wilcoxon test, and the Kruskal-Wallis test was used for more than two groups. We employed principal coordinate analysis (PCoA) based on the Bray-Curtis distance to assess the gut microbiota of different groups, which was calculated using USEARCH. The principal component analysis (PCA) was based on the Aitchison distance measure between CLR transformed sample abundance vectors (Gloor et al., 2017). We performed PERMANOVA analysis using the “adonis” function from the “vegan” R package (version: 3.5.3). The visualization of the relative abundance at the phylum and species levels was accomplished using the “ggplot2” package (version: 3.5.3). Statistical Analysis of Metagenomic Profiles (STAMP) software was used to examine significantly different bacteria between groups using Wilcoxon test (Parks et al., 2014). We used PStrain developed by the team of Shuaicheng Li to track microbial transmission at the strain level (Wang et al., 2021a). We used ImageGP^4^ to visualize the heatmap (Chen et al., 2022). All the p values were adjusted by false discovery rate (FDR), and q-values <0.05 were considered as statistically significant.

## Results

### Characteristics of cohort and sequencing data

To examine the microbiota development trajectory of mothers and infants and explore mother-infant gut microbiome transmission, we enrolled 34 CS mother-infant pairs and downloaded a corresponding Finnish dataset. A total of 107 samples were sequenced for 34 pairs. We included 10 and 34 samples of infants collected at 1 week (I1week) and 3 months (I3months) after birth. Only 10 infant samples in the first week were sequenced due to the low microbial content in feces. A total of 29 and 34 maternal samples were included, which were collected during the last week of pregnancy (M1week) and 1 month after delivery (M1month). The demographic information of the participants is presented in Table 1.

**TABLE 1:** Clinical characteristics of mothers and infants.

Variables	
Maternal age (years)	31.44 ± 3.174
Pregnancy Weight (kg)	64.176 ± 6.224
Prenatal BMI (kg/m^2^)	25.308 ± 2.055
Gestational age (days)	261.79 ± 8.800
Birth weight (g)	2811.91 ± 430.092

### Gut microbiome profile of mothers and infants in early life

**FIGURE 1:**
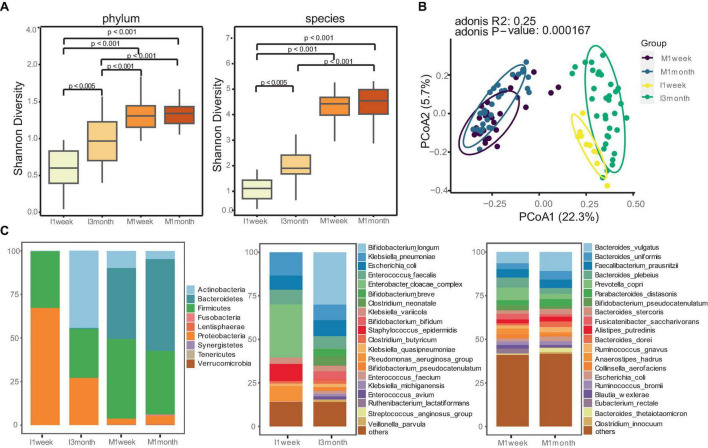
Distinct gut microbiome communities of mothers and infants. **(A)** Comparison of alpha diversity between mothers and infants at phylum and species level. The differences between two groups were assessed using the Wilcoxon test, and the Kruskal-Wallis test was used for more than two groups. **(B)** Principal coordinate analysis (PCoA) analysis was performed using the Bray-Curtis index to estimate similarities between microbial communities of mothers and infants. PERMANOVA with Adonis was used to calculate differences. **(C)** Relative abundances of gut microbiomes for mothers and infants at phylum and species level.

The α-diversity index was used to estimate microbial richness. Maternal feces showed significantly higher diversity compared to infants, both at the phylum and species level (*p* < 0.001, Figure 1A). Similarities between microbial communities were estimated using PCoA. The Bray-Curtis index and Aitchison distance were used to compare distances between groups, and PERMANOVA was used to calculate differences. The PCoA and PCA indicated a significant separation in microbiota composition between mothers and infants (*p* < 0.001, Figures 1B; *P* = 0.001, Supplementary Figure 1).

Given the significant differences in the gut microbiotas between mothers and infants, we attempted to understand how microbiota composition and developmental trajectories change. Maternal communities were dominated by species from the *Firmicutes* (40.7%) and *Bacteroidetes* (47.1) phyla, whereas infants had a higher proportion of Proteobacteria (67.1%) at 1 week and *Actinobacteria* (44.2%) at 3 months (Figure 1C). We confirmed this observation with species-level profiles (Figure 1C). At 1 week, infant samples were dominated by *Enterobacter cloacae complex, Klebsiella pneumoniae, Escherichia coli, Staphylococcus epidermidis, and Pseudomonas_aeruginosa_group.* These bacteria, commonly associated with hospital environments, accounted for 65.8% of the total microbiota composition. At 3 months, infants were enriched with *Bifidobacterium* (41.3%), including *Bifidobacterium longum* (30.0%), *Bifidobacterium breve*, *Bifidobacterium bifidum, Bifidobacterium pseudocatenulatum*. Statistical analysis revealed that *Bifidobacterium longum* was the most enriched species in the gut of infants at 3 months compared to 1 week (*p < 0.05*, Supplementary Figure 2). In contrast, maternal communities were stable and dominated by *Bacteroides*, such as *Bacteroides vulgatus, Bacteroides uniformis, Faecalibacterium prausnitzii, Bacteroides plebeius, Prevotella copri*.

### Microbiome transmission from mother to infant at strain-level

We used PStrain to track mother-infant microbiome transmission at the strain level. Pstrain (Wang et al., 2021a) is an algorithm developed by the team of Shuaicheng Li at the City University of Hong Kong. PStrain maps reads to MetaPhlAn3 marker genes based on single nucleotide variations (SNV) and infers the strain’s genotype and abundance. The strains obtained from all samples were then clustered, and the abundance of strain clusters in each sample was calculated. The cluster names represent the corresponding strains. Finally, PStrain visualized the strain proportion alteration and tacked the microbial engraftment from mother to infant. PStrain adopts an optimization strategy to profile strains iteratively to address the next-generation sequencing bias. Moreover, PStrain introduces the second-order genotype frequency as the genotype frequency at every two adjacent loci. PStrain reduces the possibility of different strain combinations resulting in the same genotype frequency patterns. In our study, If the strain genotype in the infant sample was the same as the strain genotype in the mother, we could determine the strain transmitted from the mother.

**FIGURE 2:**
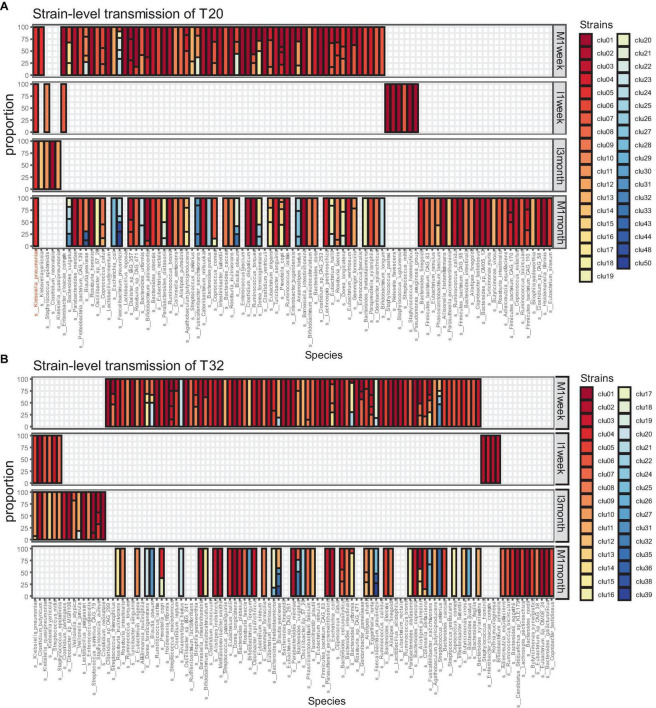
Microbiome transmission from mother to infant at strain level in T20 **(A)** and T32 **(B)**. The bar plot represents the strain proportion of each species. The different color of bar plot reflects different strains. **(A)** In the family of T20, only one strain of *Klebsiella pneumoniae* was transmitted from the mother. **(B)** In the family of T32, no stains were transmitted from the mother.

We collected samples from 34 CS infants and their mothers at different time points. Excluding missing samples, we included 32 mother-infant pairs. We used numbers to represent the mother-infant pairs included in our study, such as T20 and T32. For 32 of those families, eight pairs were sampled at four time points (I1week, I3months, M1week, and M1month). A total of 20 pairs were sampled at three time points. A total of four pairs were sampled at two time points (I3months, M1month). Our analysis showed that strain transmission from mother to CS infant was rare. Most pairs (65.6%, 21/32) did not exhibit any strain transmission, such as T32 (Figure 2B). Only 11 (34.4%, 11/32) pairs showed strain transmission events, such as T20 (Figure 2A). Moreover, in pairs where strain transmission occurred, most mothers showed transmission of only one strain to the infants (81.8,9/11). Finally, a total of 14 strain transmission events were observed in 11 families. The 14 strains belonged to seven species. Strains from *Klebsiella pneumonia* were transmitted most frequently (57.1%, 8/14). The other strains were transferred only once, which included *Bifidobacterium longum*, *Blautia wexlerae*, *Escherichia coli*, *Klebsiella variicola*, *Eggerthella lenta* and *Ruminococcus gnavus*.

To further analyze the mother-infant microbial transmission, we downloaded Finnish mother-infant pair data. The original FASTQ file was downloaded from the BioProject database on the NCBI (PRJNA475246). Then, we used the PStrain to analyze and visualize the strain transmission from mother to infant. Yassour et al. (2018) collected stool samples from a cohort of 44 infants and their mothers and performed whole-genome metagenomic sequencing. For 33 of those families, they collected samples at three time points from the mothers (gestational week 27, delivery, and 3 months after delivery) and at five time points from the infants (at birth, 2 weeks of age, and 1, 2, and 3 months of age). The remaining 11 families had only three samples: maternal samples collected at gestational week 27 and delivery and child meconium samples. We employed the PStrain to analyze the strain transmission in 33 mother-infant pairs. One family was excluded because only infant samples were collected, and the samples from mothers were not collected. As a result, we included a total of 32 mother-infant pairs (28 VD, 4 CS) for further analysis.

**FIGURE 3:**
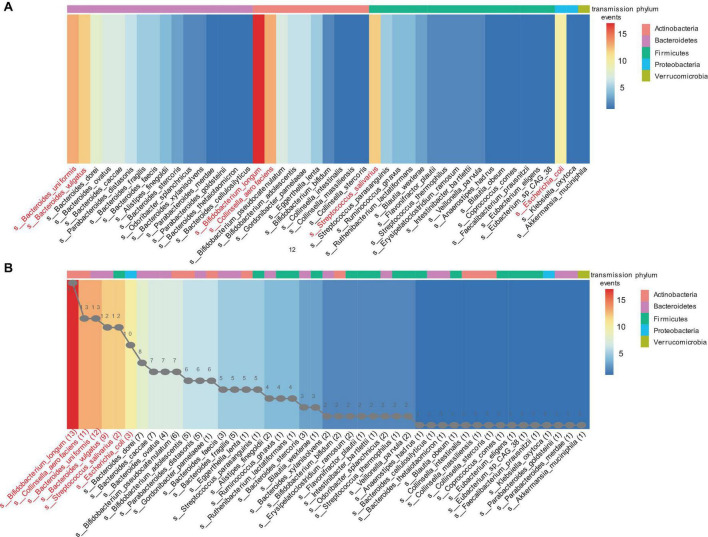
The mother-infant transmission events in the Finish cohort. A strain transmission event is defined as the transfer of a strain from mother to infant for one time. **(A)** Transmission events in each phylum. **(B)** Sorting by the number of transmission events. The line graph in panel **(B)** represents the number of transmitted strains for each corresponding species. For example, 13 strains of *bifidobacterium longum* were transmitted to infants 17 times across 30 mother-infant pairs. The heatmap ranges from blue to red, indicating an increasing number of transmission events. The different colored blocks at the top of the heatmap represent the phylum to which the species belongs.

Strain transmission was observed in 30 infants (93.4%, 30/32), including 28 VD infants and two CS infants. All VD infants received transmitted strains from their mothers. The two CS infants received three and five strains transmitted from their mothers. The other two CS infants did not exhibit any strain transmission. Finally, 193 strain transmission events were observed, including 131 strains and 45 species. The highest frequency of strain transmission belonged to the *Bacteroidetes* phylum, accounting for 40.4% (78/193) of the total transmission. These transmissions were observed across 22 families. Strains from *Actinobacteria* phylum accounted for 30.6% (59/193) of the transmissions, which were distributed among 28 families. Strains from *Firmicutes* phylum accounted for 22.8% (44/193) of the transmissions, spanning 23 families. In contrast, *Proteobacteria* phylum exhibited the lowest frequency of strain transmission, amounting to 5.7% (11/193) of the total and occurring in 10 families. Additionally, a single transmitted strain was identified from *Akkermansia muciniphila*, belonging to the *Verrucomicrobia* phylum (Figure 3A). At the species level, strains from the *Bifidobacterium longum* accounted for the highest frequency of transmission, representing 8.8% (17/193) of the total strain transmission, followed by *Bacteroides uniformis* (6.7%. 13/193), *Collinsella aerofaciens* (6.7%, 13/193), *Bacteroides vulgatus* (6.2%, 12/193), *Streptococcus salivarius* (6.2%, 12/193), and *Escherichia coli* (5.2%, 10/193) (Figure 3B).

## Discussion

We sequenced microbiomes from 34 CS newborns and their mothers to determine the microbiota development trajectory of mothers and infants and explore mother-infant gut microbiome transmission by comparing with a corresponding Finnish dataset.

We compared the gut microbial diversity and composition of mothers and infants. Our study revealed that mothers and infants possessed distinct gut microbial compositions and developmental patterns. The maternal communities were dominated by species from *Firmicutes* and *Bacteroidetes* before and after delivery. This gut microbial pattern was consistent with that of the gut of a healthy Chinese adult. Yang et al. (2020) sampled the gut microbiotas of 1,479 pregnant women of Chinese origin from 9 weeks of gestation to antepartum and compared them to those of 1,048 age-matched non-pregnant women. The gut microbiota of pregnant women displayed a similar overall structure to that of normal adults. The gut microbiota of adults exhibits a considerable degree of stability and resilience, allowing it to demonstrate the tendency of restoration to original composition despite encountering certain external pressures (David et al., 2014; Palleja et al., 2018; Derrien et al., 2019). In contrast, the structure of the infant gut microbial composition is highly variable but also follows predictable patterns in the early stages of life (Bäckhed et al., 2015; Lim et al., 2015). The gut microbiome of infants ranged from an anaerobic community dominated by *Enterobacteriaceae* and *Enterococcaceae* in the first week of life to the establishment of *Bifidobacterium* as the dominant gut microbe by 3 months of age.

Subsequently, we explored microbial transmission from mother to infant. Our study comprised 32 CS infants, and transmission occurred in only 11 pairs. A total of 14 strain transmission events were observed in 11 pairs, comprising 14 strains from 7 species. In the Finnish data, strain transmission was observed with all VD infants. A total of 193 strain transmission events were observed, comprising 131 strains and 45 species. Our analysis showed that strain transmission from mother to infant is rare in CS infants. CS delivery disrupts the mother-to-infant transmission of microbiota, as demonstrated by previous studies (Korpela et al., 2018; Shao et al., 2019; Mitchell et al., 2020; Feehily et al., 2023). Korpela et al. (2018) analyzed 25 infants and six 2- to 10-yr-olds born via CS. Only six of the 15 CS infants had any species overlap with the mothers to allow for SNV analysis and did not share a single strain. The mother-to-infant strain transmission in VD was 0.87 and 0 in CS. Performing strain transmission analysis from 178 mother-baby dyads, Shao et al. (2019) found that maternal strain transmission occurred mainly in VD infants (74.39%) during the neonatal period, with a high rate of transmission compared to those delivered by C-section (12.56%). Feehily et al. (2023) found that the diversity of species shared through vaginal births was greater than those transferred during CS, with only 7 different shared species observed with CS compared to 26 with vaginal births.

Infants delivered via C-section are not exposed to the vaginal and fecal microbiome compared to infants delivered via vagina at birth; therefore CS infants bypass the first colonization of pioneering gut microbes. This may be one of the reasons for the lack of mother-infant microbiome transmission in CS infants. Some studies have examined the restoration of the disputed microbiome of CS infants by exposure to the maternal microbes, including vaginal and maternal microbiome. This process is known as mother-infant microbiome seeding. A sterile gauze is inserted into the mother’s vagina before C-section delivery and then wiped over the body of four CS infants. The gut, mouth, and skin communities of CS infants were enriched with vaginal bacteria, similar to those in VD infants (Dominguez-Bello et al., 2016). This partially restored the microbiome development of CS infants over the first month of life. In another study, 30 infants delivered by Cesarean section were swabbed with maternal vaginal gauze immediately after birth. The microbiomes of exposed CS infants aligned more closely with those of vaginally born babies (Song et al., 2021). Vaginal seeding increases mother-infant microbiota transmission and reduces the Shannon index of the skin and stool microbiota (Mueller et al., 2023). However, subsequent studies suggested that vaginal seeding does not correct the CS-induced microbiota imbalance. Wilson et al. (2021) orally administered a 3 mL solution of either maternal vaginal microbes (CS-seeded, *n* = 12) or sterile water (CS-placebo, *n* = 13) to CS infants. No differences in microbial composition or function between the two groups were noted at 1 or 3 months. The CS-seeded infants displayed the characteristic signature of low *Bacteroides* abundance, as expected with normal CS infants. Moreover, other approaches to maternal-child microbial seeding have also been proposed, such as maternal fecal microbiota transplantation (FMT). CS infants who orally received maternal FMT within 2 h of birth showed restoration of microbiota resembling that of vaginally born infants from 2 days until 3 months of age compared to C-section infants who were not treated. In particular, maternal FMT corrected the persistent lack of *Bacteroides* spp. (Korpela et al., 2020). Despite the growing interest in maternal-child microbial seeding, many controversies still surround the process, including safety concerns, regarding the rationale and regulatory issues (Hourigan et al., 2022).

This study had several limitations. First, we only assessed the microbiota composition of CS infants and their mothers. In further studies, it is necessary to collect sufficient VD infant samples for analysis. Second, antibiotics can influence the microbiota. All mothers who underwent CS used antibiotics to prevent infection after surgery. However, we did not collect antibiotic use data of infants. It is important to collect this information and analyze the impact of antibiotics in future studies. We only collected samples of infants 1 week and 3 months after birth, and samples of mothers before pregnancy and 1 month after pregnancy. The samples of mothers and infants should be collected at more matched time points to observe the mother-infant transmission.

## Conclusion

In the early stages of life, we analyzed the gut microbial development of mothers and infants and explored the mother-infant strain transmission compared with a corresponding Finnish dataset by applying whole-genome shotgun metagenomic analysis. We found that mothers and infants possessed distinct gut microbial compositions and developmental patterns. In addition, CS disrupted the mother-infant strain transmission. The intestinal strain transmission from mother to infant was rare in CS infants. In the future, large-scale, high-resolution, and long-term cohort studies are necessary to explore the impact of delivery mode on the mother-infant strain transmission.

## Data availability statement

The datasets presented in this study can be found in online repositories. The names of the repository/repositories and accession number(s) can be found below: https://db.cngb.org/search/project/CNP0003746/, CNP0003746.

## Ethics statement

The studies involving humans were approved by the Ethical Committee of the West China Second Hospital of Sichuan University. The studies were conducted in accordance with the local legislation and institutional requirements. Written informed consent for participation in this study was provided by the participants’ legal guardians/next of kin. The animal study was approved by Ethical Committee of the West China Second Hospital of Sichuan University. The study was conducted in accordance with the local legislation and institutional requirements. Written informed consent was obtained from the individual(s), and minor(s)’ legal guardian/next of kin, for the publication of any potentially identifiable images or data included in this article.

## Author contributions

RY: Data curation, Formal analysis, Investigation, Methodology, Visualization, Writing—original draft, Writing—review and editing. YNW: Data curation, Formal analysis, Methodology, Writing—review and editing. ZYY: Resources, Software, Writing—review and editing. ZYS: Data curation, Investigation, Writing—review and editing. YS: Data curation, Investigation, Writing—review and editing. JY: Data curation, Investigation, Writing—review and editing. SLH: Data curation, Investigation, Writing—review and editing. ZCZ: Formal analysis, Methodology, Writing—review and editing. YLH: Conceptualization, Data curation, Investigation, Writing—review and editing. QC: Conceptualization, Data curation, Investigation, Writing—review and editing. WTP: Conceptualization, Project administration, Supervision, Writing—review and editing. XWL: Conceptualization, Project administration, Supervision, Writing—review and editing.

## Footnotes

1
https://www.shbio.com/products/3033

2
http://huttenhower.sph.harvard.edu/kneaddata

3
http://huttenhower.sph.harvard.edu/biobakery

4
https://www.bic.ac.cn/ImageGP/
