# Supplementary material for: Inspecting mother-to-infant microbiota transmission: disturbance of strain inheritance by cesarian section
Source: Front Microbiol. 2024 Feb 29;15:1292377. doi: 10.3389/fmicb.2024.1292377 (PMC10937581; doi:10.3389/fmicb.2024.1292377)
Supplement: Supplementary file 1 [file Data_Sheet_1.docx]

**Supplementary Figures**


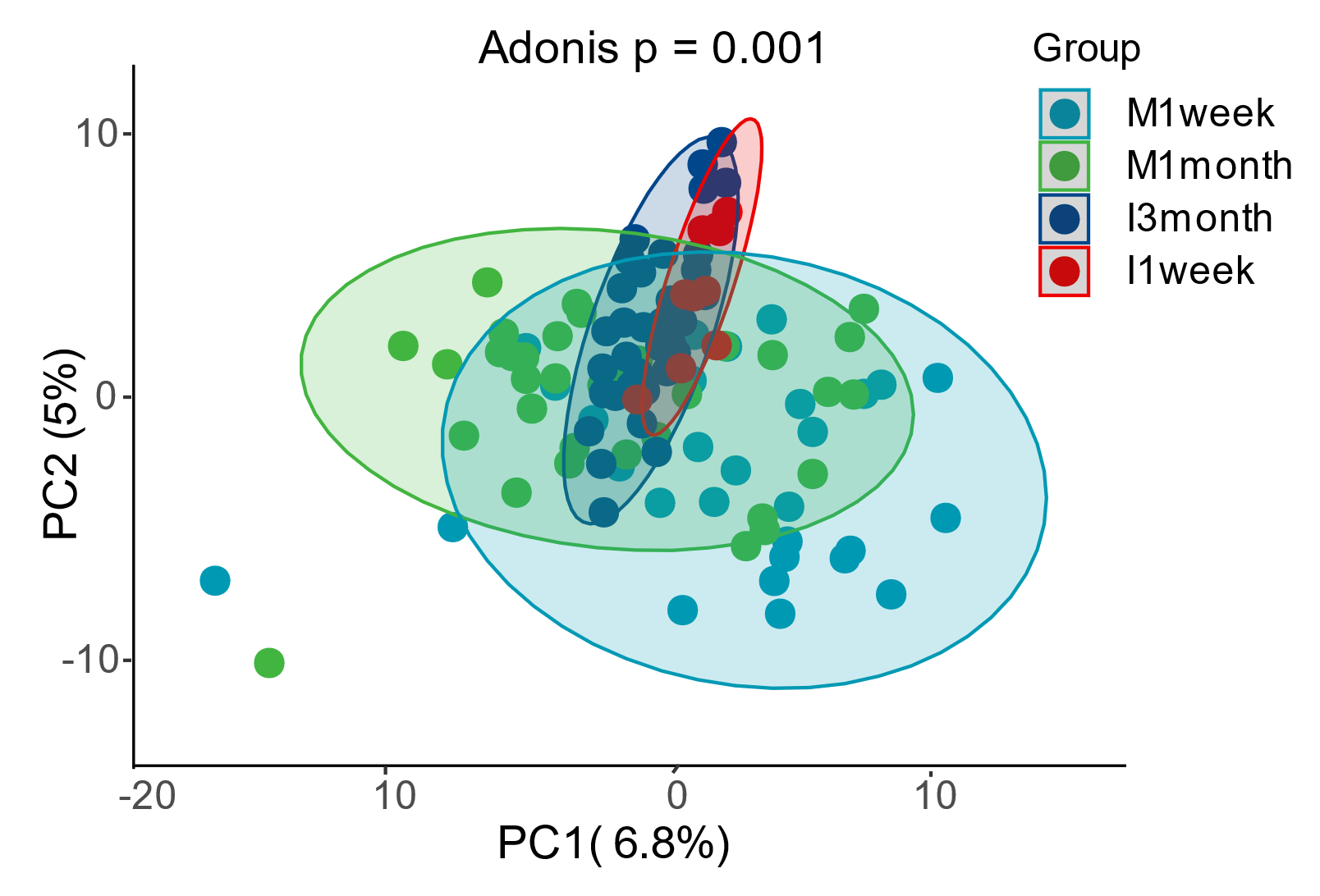


**Figure S1.** PCA analysis based on the Aitchison distance to estimate similarities between microbial communities of mothers and infants. PERMANOVA with adonis was used to calculate differences.


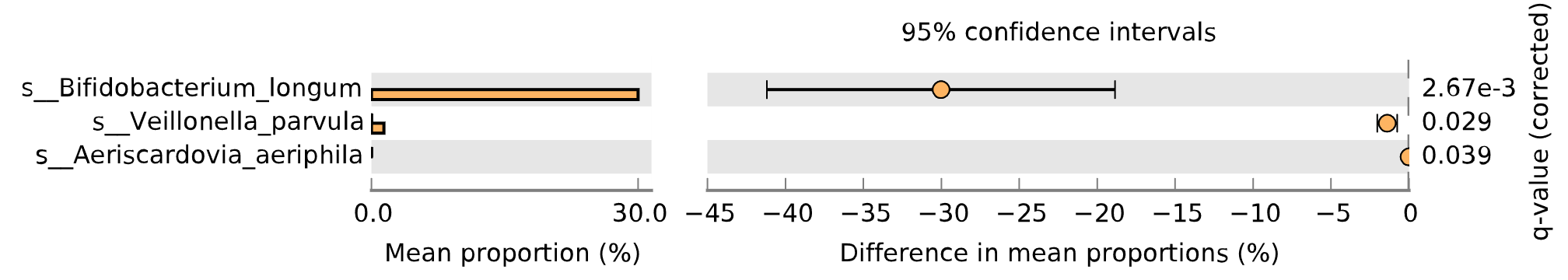


**Figure S2.** Statistically significant different species in the gut microbiota of infant at 3 months compared to 1 week. STAMP software was used to conduct significantly different bacteria between groups with Wilcoxon test.
